# Two-component system VraSR senses host defense peptide PMAP-36 to promote *Streptococcus suis* pathogenicity through regulating *accD* expression

**DOI:** 10.1186/s13567-026-01858-z

**Published:** 2026-10-22

**Authors:** Yandi Pan, Yi Lu, Tao Zhang, Zichen Hu, Hongdou Liu, Bo Su, Rui Lei, Tingting Zhang, Yaru Zhang, Xuefeng Cao, Zhiwei Li, Rendong Fang, Lianci Peng

**Affiliations:** Joint International Research Laboratory of Animal Health and Animal Food Safety, College of Veterinary Medicine, Southwest University, No. 2 Tiansheng Road, Beibei District, Chongqing, 400715 China

**Keywords:** *Streptococcus suis*, PMAP-36, virulence, two-component system, fatty acid synthesis

## Abstract

*Streptococcus suis* (*S. suis*) is an important zoonotic pathogen that causes severe infections in humans and animals worldwide. Upon infection, the host innate immune system produces host defense peptides (HDPs) to resist *S. suis* invasion. PMAP-36, a porcine-derived HDP, has been shown to fail to kill *S. suis*, suggesting that this pathogen has evolved strategies to evade PMAP-36-mediated immune defense. However, the exact mechanism by which *S. suis* responds to PMAP-36 remains unclear. Here we showed that PMAP-36 significantly enhanced *S. suis* adhesion, cytotoxicity, and virulence gene expression. The two-component system (TCS) VraSR was essential for PMAP-36 to promote *S. suis* pathogenicity. We demonstrated that PMAP-36 directly bound to the sensor kinase VraS. Further transcriptomic analysis revealed that VraSR sensed PMAP-36 to regulate fatty acid biosynthesis, in which a key gene *accD* expression was significantly downregulated in both PMAP-36-treated Δ*vraS* and Δ*vraR* mutant strains. Deletion of *accD* attenuates *S. suis *in vivo pathogenicity, including reduced bacterial loads, alleviated organ damage, and decreased inflammatory response. Additionally, VraSR-mediated *accD* expression was critical for *S. suis*-induced cytotoxicity and inflammatory response, as deletion of *vraS* or *vraR* fails to reduce inflammatory cytokines production due to sustained high *accD* expression. This study reveals VraSR-mediated virulence regulatory mechanism in response to HDP activation, which provides new insight into the development of novel strategies to combat *S. suis* infection.

**Supplementary Information:**

The online version contains supplementary material available at https://doi.org/10.1186/s13567-026-01858-z.

## Introduction

Host defense peptides (HDPs), known as antimicrobial peptides (AMPs), are one of the most important components in the innate immune system against microbial infection [1]. HDPs from vertebrates mainly contain two classes including defensins and cathelicidins, which are produced as prepropeptides and then cleaved to mature peptides. Upon infection, these peptides are largely produced by different types of cells including epithelial cells and neutrophils to kill pathogens through various mechanisms, directly killing at high local concentrations or indirectly modulating immune response [1]. They exert anti-inflammatory or pro-inflammatory activities depending on the phase of infection [2, 3]. To date, AMPs have emerged as a promising strategy to address the antibiotic resistance crisis. Although bacterial resistance against AMPs is not widely spread in pathogens, some pathogens have evolved different mechanisms to resist AMPs killing, such as secreting proteases to degrade AMPs, harboring acquired antimicrobial resistance genes and modifying cell surface components to reduce AMPs’ binding and penetration as well as interacting with bacterial outer membrane protein to promote biofilm formation [4–8]. Therefore, elucidating the mechanism by which bacteria evade AMPs will contribute to identify novel and effective antibacterial targets.

*Streptococcus suis* (*S. suis*) as an important zoonotic pathogen has been characterized to evade the host immune defense, such as cleaving AMPs, avoiding phagocytic killing, interacting with complement factor, and suppressing the host’s oxidative response [9–12]. Two-component systems (TCSs) as the important regulatory systems responding to external environmental stimuli including AMPs, play a crucial role in modulating *S. suis* immune evasion or virulence. The human-derived HDPs have been reported to activate TCSs, thereby promoting bacterial pathogenicity [13, 14]. Diverse TCSs have been identified to contribute the virulence or pathogenicity of *S. suis*, including VraSR, NisKR, 1910HK/RR, Ihk/Irr, CiaRH, SalK/SalR, and VirR/VirS [15–23]. Among these TCSs*,* VraSR is one of the most extensively characterized TCSs, which is involved in multidrug resistance, thus plays a critical role in combating AMPs [16, 17]. Despite the well-documented biological functions of VraSR, the host-derived signals that activate this system remain poorly defined, and its downstream regulatory network needs to be further characterized. Our previous study has shown that porcine-derived HDP PMAP-36 was not able to effectively kill *S. suis* [24], indicating that *S. suis* escapes its killing. However, the exact mechanism how *S. suis* responds to PMAP-36 remains unclear. This study focuses on the host-derived signal molecule PMAP-36-mediated *S. suis* pathogenicity and the *accD*-mediated fatty acid metabolic pathway for in-depth investigation.

In this study, we demonstrate that PMAP-36 binds to the sensor VraS of *S. suis* and activates VraSR, which subsequently promotes virulence genes expression and enhances bacterial cytotoxicity towards the host cells. Further transcriptome analysis reveals that VraSR senses PMAP-36 to regulate fatty acid metabolism and biosynthesis. The in vitro and in vivo assays confirm that the key fatty acid synthesis gene *accD* plays a critical role in the pathogenicity of *S. suis*. Our findings highlight the detailed role of VraSR in promoting *S. suis* pathogenicity via regulating *accD* expression in response to PMAP-36, serving as an essential strategy for *S. suis* to sense HDPs and exploit HDPs to facilitate its invasion.

## Materials and methods

### Mice

Wild-type (WT) C57BL/6 mice were purchased from Hunan SJA Laboratory Animal Co., Ltd. All mice were maintained under specific pathogen-free conditions and used at 8–10 weeks of age. This study was approved by the Institutional Animal Care and Use Committee (IACUC) of Southwest University, Chongqing, China (IACUC-20241031-02).

### Bacterial strains and mutant strains construction

*Streptococcus suis* serotype 2 strain SC19 was kindly provided by Professor Xiangru Wang from Huazhong Agricultural University, Wuhan, China. The knockout mutants Δ*vraS*, Δ*vraR*, Δ*accD*, and Δ*vraR*Δ*accD* were constructed in the SC19 background as described below. Bacteria were cultured in tryptic soy broth (TSB; Beijing Luqiao Technology Co., Ltd., China) or tryptic soy agar (TSA) supplemented with 10% fetal bovine serum (FBS; Zhejiang Tianhang Biotechnology Co., Ltd., China) at 37 °C until mid-logarithmic phase before being used. *Escherichia coli* (*E. coli*) BL21 strain was cultured in Luria–Bertani (LB) broth (HopeBio, Qingdao, China) or on LB agar plates (1.5% agar).

The Δ*vraS*, Δ*vraR*, Δ*accD*, and Δ*vraR*Δ*accD* mutant strains were constructed via homologous recombination using the thermosensitive suicide vector pSET4s. Briefly, the upstream and downstream homologous arm fragments of the target genes were amplified by polymerase chain reaction (PCR) using specific primers. The amplified products were cloned into the corresponding restriction sites of the pSET4s vector using T4 DNA ligase (Vazyme, China) to generate target genes deletion vectors. Subsequently, these recombinant vectors were electroporated into SC19 competent cells and single-crossover recombinants were selected in TSA containing spectinomycin (100 μg/mL; Macklin, Shanghai, China) at 28 °C. The obtained colonies were then inoculated onto antibiotic-free TSA plates and incubated at 37 °C to induce double-crossover homologous recombination via the thermosensitive nature of the vector, thereby generating the isogenic mutant strains. Finally, the deletion of the target genes was confirmed by PCR amplification and DNA sequencing. All primers for mutant strains construction are shown in Additional file 1.

### Cell culture

Human cerebral microvascular endothelial cells (hCMEC/D3; Jennio Biotech, Guangzhou, China) and mouse monocyte-macrophage leukemia cells (RAW264.7; JNO-M0201, Jennio Biotech) were cultured in Dulbecco’s modified Eagle medium (DMEM; Gibco) supplemented with 10% fetal bovine serum (FBS; Excell, Jiangsu, China). Mouse peritoneal macrophages were cultured in Roswell Park Memorial Institute 1640 medium (RPMI 1640, Vivacell, Shanghai, China) supplemented with 10% FBS (BBI, Sangon Biotech, Shanghai, China). All cells were maintained at 37 °C with 5% CO_2_.

### The growth curves of bacterial strains

Wild-Type (WT) and mutant bacterial strains were incubated with different concentrations of PMAP-36 (5 μM and 10 μM) for 0, 1, 2, 3, 4, 5, 6, 9, and 12 h. After incubation, bacterial suspensions were serially 10-fold diluted and plated onto TSA plates. Then, the plates were incubated at 37 °C for 12–16 h, and colony-forming units (CFUs) were counted.

### Quantitative real‑time polymerase chain reaction (RT-qPCR)

WT and mutant bacterial strains (1×10^6^ CFU/mL) were incubated with PMAP-36 (5 μM or 10 μM) for 6 h. Then, bacterial RNA was extracted using a Bacterial RNA Kit (Omega, USA). Next, cDNA was synthesized according to the manufacturer’s instructions of the reverse transcription kit (Accurate Biology, Hunan, China), and RT-qPCR was performed under the following conditions: 95 °C for 3 min; 40 cycles of 95 °C for 5 s and 60 °C for 30 s; followed by a melting curve program of 95 °C for 5 s, 60 °C for 5 s, and 95 °C for 2 min. The results were analyzed using the 2⁻^ΔΔCT^ method. All primers for gene expression are shown in Additional file 2.

### Cell adhesion assay

Bacterial strains (1 × 10^6^ CFU/mL) were treated with or without PMAP-36 (2.5 μM) for 6 h and then harvested by centrifugation. After washing to remove residual peptides, the bacteria were resuspended in DMEM supplemented with 10% for being used as the infection inoculum. Next, hCMEC/D3 cells were infected with bacteria at a multiplicity of infection (MOI) of 10 at 37 °C with 5% CO_2_. After 3 h of infection, cells were washed three times with phosphate-buffered saline (PBS), and 300 μL of sterile ice‑cold water was added to each well, followed by incubation at 37 °C for 15 min to lyse cells for adherent bacteria quantification [25]. Subsequently, 50 μL aliquots from each well were serially 10‑fold diluted and plated on TSA plates, followed by incubation at 37 °C for 12–16 h. Finally, CFUs were counted and the bacterial adhesion rate was calculated as follows: adhesion rate (%) = (the number of adherent bacteria/the initial number of inoculated bacteria) × 100%.

### Hoechst/propidium iodide (PI) staining

The bacterial infection inoculum was prepared as described above. Then, RAW264.7 cells were infected with bacteria at an MOI of 10 for 6 h. After infection, cells were washed three times with PBS to remove nonadherent bacteria. Next, cells were stained with Hoechst/PI (KeyGEN BioTECH, Jiangsu, China) in the dark accoring to the manufacturer’s instructions. After staining, cells were wanshed with PBS and anti-fade mounting medium was added to cells. Finally, cells were observed by fluorescence microscopy.

### Protein expression and purification

The gene sequences of VraS were amplified using specific primers and PCR products were cloned into pGEX‑4 T‑1. The recombinant plasmid was transformed into *E. coli* BL21(DE3) strain, followed by overnight culture at 37 °C with shaking at 180 rpm. Then, the culture was diluted at 1:100 into 400 mL of LB containing ampicillin (50 μg/mL; Macklin, Shanghai, China), and incubated to mid-logarithmic at 37 °C with shaking until an OD of 0.8 at 600 nm. Subsequently, protein expression was induced by adding 0.3 mM isopropyl‑β‑D‑thiogalactopyranoside (IPTG; BBI, Shanghai, China), followed by overnight incubation at 16 °C with shaking. After incubation, bacteria were harvested by centrifugation at 8000 rpm for 10 min at 4 °C and resuspended in 20 mL of PBS, followed by sonication on ice to obtain bacterial lysate. After sonication, the lysate was centrifuged at 8000 rpm for 10 min at 4 °C, and the supernatant was collected and filtered through a 0.22 μm filter on ice. Finally, protein purification was performed using GST-tag Purification Resin (Beyotime, China) and Ni Elite Beads (Vazyme, Nanjing, China). The purified protein was stored at −80 °C. All primers for protein expression are shown in Additional file 1.

### Cellular thermal shift assay (CETSA)

To determine the interaction between PMAP-36 and VraS, CETSA was performed. Recombinant GST-tagged VraS protein (50 μg/mL) or GST were incubated with either PMAP-36 (100 μM), H_2_O or polymyxin B (as a negative control) at 25 °C for 20 min. The mixtures were then heated in a gradient thermal cycler at 25, 37, 45, 52, 57, 62, 67, and 72 °C for 5 min each, followed by immediate immersion in an ice bath for 3 min. Finally, the samples were analyzed by western blotting using an anti-GST tag polyclonal antibody (Proteintech, Wuhan, China) to detect the VraS protein.

### Drug affinity responsive target stability assay (DARTS)

To determine the interaction between PMAP-36 and VraS, DARTS assay was performed. Recombinant GST-tagged VraS protein (50 μg/mL) or GST were incubated with either PMAP-36 (100 μM) or H_2_O (as a negative control) at room temperature for 30 min. Subsequently, Pronase E at concentrations of 1, 2, and 5 μg/mL was added to each sample, and the mixtures were incubated at room temperature for 20 min. Finally, the samples were analyzed by western blotting using an anti-GST tag polyclonal antibody to detect the VraS protein. Data are presented from three biological replicates (*n* = 3). Student’s *t*-test was used to analyze the statistical differences for comparisons between two groups.

### GST affinity isolation assay

Purified GST-VraS recombinant protein and GST alone were respectively incubated with FITC-labeled PMAP-36 in 500 μL of NP-40 lysis buffer for 2 h, followed by addition of GST resin and further incubation at 4 °C for 4 h. After centrifugation at 2200 rpm for 3 min at 4 °C, the samples were washed ten times with PBS and then visualized under a fluorescence inverted microscope.

### Molecular docking and molecular dynamics simulations

The three-dimensional structures of PMAP-36 and VraS (GenBank: ARL69388.1) were predicted using AlphaFold3, and the top-ranked structures based on pLDDT scores were selected for subsequent analysis. Protein–protein docking was performed using the local version of HDOCKlite v1.1 with a global docking strategy. The docking models were ranked according to docking and confidence scores, and the model with the highest confidence and favorable docking score was selected for further analysis. The binding free energy of the PMAP-36-VraS complex was subsequently predicted in the HawkDock server. Protein–protein interactions within the docking complex were analyzed using PLIP and visualized with PyMOL. The stability and conformational dynamics of the PMAP-36-VraS complex were further evaluated by molecular dynamics simulations. The docked PMAP-36-VraS complex and VraS alone were subjected to molecular dynamics simulations, and the trajectories were analyzed based on root-mean-square deviation (RMSD), radius of gyration (Rg), solvent-accessible surface area (SASA), residue-level root-mean-square fluctuation (RMSF), and hydrogen-bond formation.

### RNA-seq sample preparation

*S. suis* SC19, Δ*vraS*, and Δ*vraR* strains (1×10^6^ CFU/mL) were treated with PMAP-36 (10 μM) in 35 mL culture medium at 37 °C for 6 h, followed by centrifugation at 3000 rpm for 10 min to obtain pellets. Pellets were washed and snap-frozen in liquid nitrogen for 15 min. RNA-seq was performed by Novogene Co., Ltd. (Beijing, China). Adjusted *P* value (padj) < 0.05 and |log_2_(fold change)| > 0 were set as the screening threshold.

### Mouse infection

WT C57BL/6 mice were randomly divided into three groups: (i) PBS-treated, (ii) SC19-infected, and (iii) Δ*accD*-infected. Mice were intraperitoneally challenged with either SC19 or the Δ*accD* mutant at a dose of 1.5 × 10^8^ CFU in 0.2 mL of PBS. During infection, the clinical status and mortality of the mice were daily monitored for 7 days (total *n* = 20, each group *n* = 10). In addition, after 10 h infection, blood, liver, spleen, lung, kidney, and brain were collected to determine bacterial loads and inflammatory cytokines production (total *n* = 15, each group *n* = 5). Meanwhile, lung, spleen, kidney, and liver were fixed in 4% paraformaldehyde and embedded in paraffin for hematoxylin and eosin (HE) staining (total *n* = 9, each group *n* = 3). The stained sections were scored in a semi-quantitative and blinded manner as described previously [26]. Briefly, less than 5%, between 5 and 10%, and more than 10% infiltrated inflammatory leukocytes in the section were defined as 1, 2, and 3, respectively.

### Enzyme‐linked immunosorbent assay (ELISA)

Mouse peritoneal macrophages were infected with bacterial strains at an MOI of 10 at 37 °C for 6 h and then gentamicin was added for 18 h additional incubation. After 24 h infection, cell supernatants were collected. Inflammatory cytokines including IL-1β and IL-6 (Invitrogen, Carlsbad, CA, USA) in the cell supernantants and animal samples were determined via ELISA according to the manufacturer’s instructions.

### Statistical analysis

GraphPad Prism 10 software (San Diego, CA, USA) was used for statistical analysis. All data are presented as mean ± standard deviation (SD) and representative of three independent experiments with at least three biological replicates. Independent biological replicates served as the experimental unit for all statistical tests where *n* represents the number of biological replicates. Student’s *t*-test and one-way analysis of variance (ANOVA) followed by Tukey’s post hoc test were used to analyze the statistical differences for comparisons between two groups and more than two groups, respectively. A value of *P* < 0.05 was considered statistically significant. *P* value is shown in the Figures.

## Results

### The VraSR TCS is activated upon PMAP-36 stimulation

**Figure 1:**
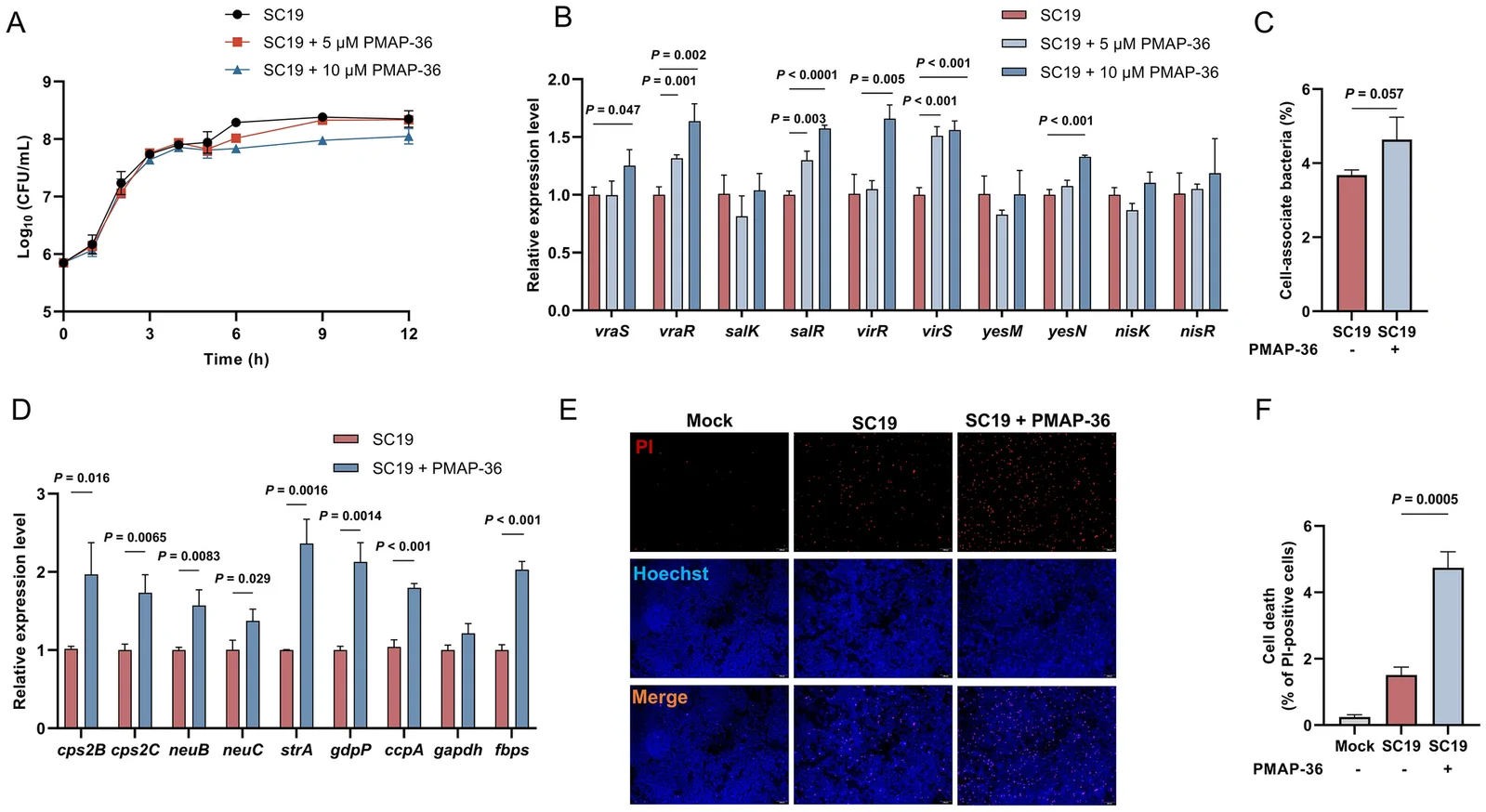
**The VraSR TCS is activated upon PMAP-36 stimulation.** **A** Growth curves of SC19 incubated with 5 μM or 10 μM PMAP-36 within 12 h. **B** TCSs gene expression of SC19 treated with or without 5 μM or 10 μM PMAP-36. **C** SC19 adhesion towards hCMEC/D3 cells after bacterial pre-incubation with or without 2.5 μM PMAP-36 for 6 h. **D** CPS synthesis and adhesion-related gene expression of SC19 treated with or without 10 μM PMAP-36 for 6 h. **E** Representative images of Hoechst/PI double staining of RAW264.7 cells infected with SC19 where bacteria have been pretreated with or without 2.5 μM PMAP-36 for 6 h. Scale bar, 100 μm. **F** Quantification of PI-positive cells in **E** using ImageJ. All data are presented as mean ± SD and representative of three independent experiments with three biological replicates (*n* = 3). Statistical analysis was performed using Student’s *t*-test.

To evaluate the impact of PMAP-36 on SC19, bacterial growth curves were firstly determined under the treatment of PMAP-36 at 5 μM and 10 μM for 12 h. The results showed that PMAP-36 treatment did not impair bacterial growth (Figure 1A). TCSs are critical signal transduction molecules that enable bacteria to sense environmental stress and maintain cellular homeostasis. To investigate whether PMAP-36 modulates TCSs in SC19, the expression of different TCSs was determined using RT-qPCR. The results revealed that PMAP-36 significantly upregulated the gene expression of multiple TCSs including *vraSR*, *salKR*, and *virRS*, but did not affect the gene expression of *yesNK* and *nisKR* (Figure 1B). Given the regulatory roles of TCSs in bacterial virulence, we subsequently assessed the impact of PMAP-36 on bacterial adhesive capacity, the expression of capsule (CPS) synthesis and adhesion-related genes, as well as its ability to induce macrophage cell death. The results showed that PMAP-36 enhanced SC19 adhesion towards hCMEC/D3 (Figure 1C). Furthermore, PMAP-36 significantly upregulated the mRNA expression of CPS synthesis genes (*cps2B*, *cps2C*, *neuB*, *neuC*) and adherence-related genes (*strA*, *gdpP*, *ccpA*, *fbps*) (Figure 1C). In addition, bacteria pretreated with PMAP‑36 triggered significantly increased PI uptake in murine macrophages, indicating enhanced macrophage cell death (Figure 1E, F). These results demonstrate that PMAP-36 promotes bacterial adhesion and cytotoxicity.

### The VraSR TCS senses PMAP-36 to regulate *S. suis* adhesion and cytotoxicity

**Figure 2:**
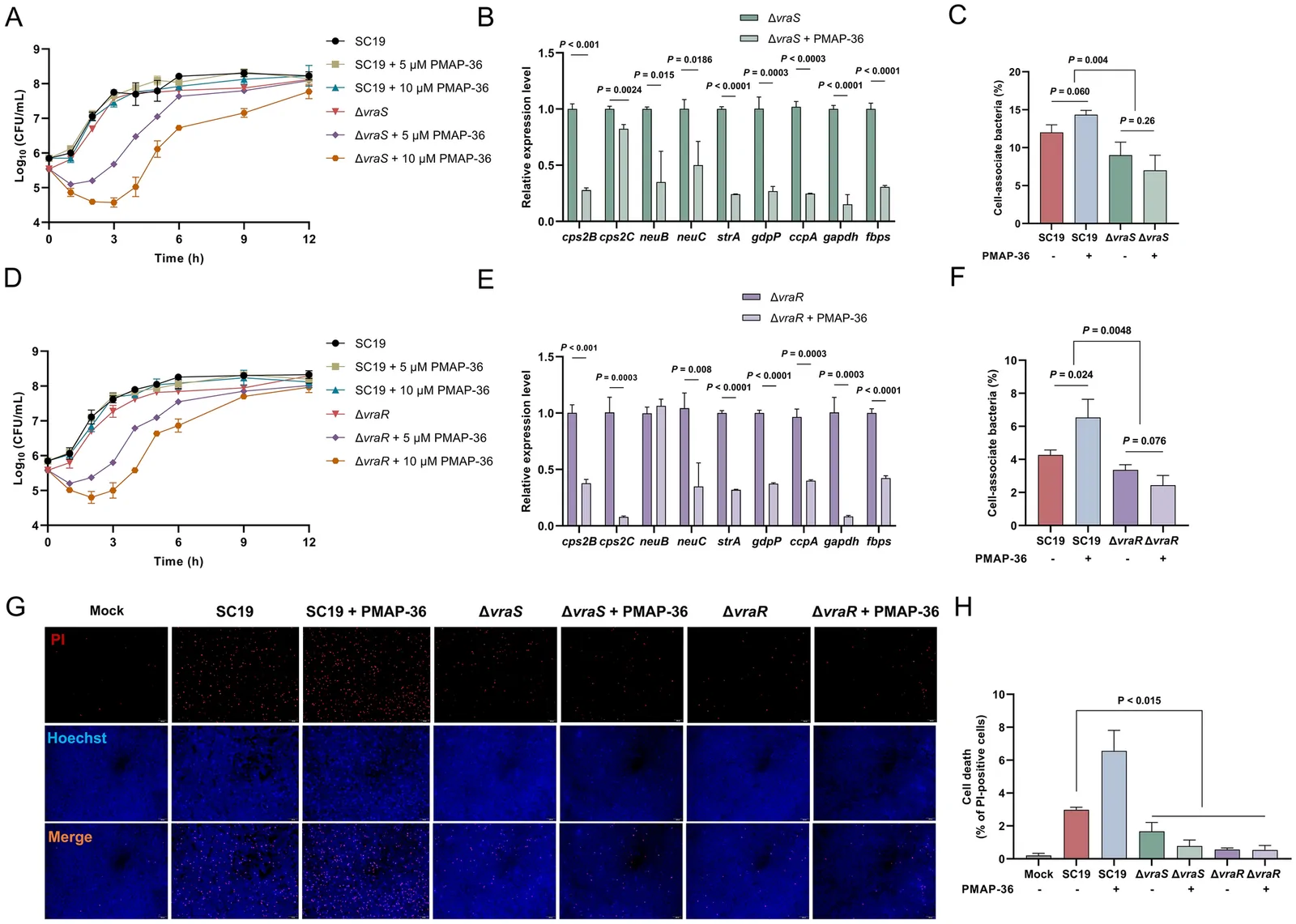
**The** **VraSR TCS** **senses PMAP-36 to regulate** ***S. suis*** **adhension and cytotoxicity**. **A** Growth curves of SC19 and Δ*vraS* treated with or without 5 μM or 10 μM PMAP-36 within 12 h. **B** CPS synthesis and adhesion-related gene expression of Δ*vraS* treated with or without 10 μM PMAP-36 for 6 h. **C** SC19 and Δ*vraS* adhesion towards hCMEC/D3 cells after bacterial pre-incubation with or without 2.5 μM PMAP-36 for 6 h. **D** Growth curves of SC19 and Δ*vraR* treated with or without 5 μM or 10 μM PMAP-36 within 12 h. **E** CPS synthesis and adhesion-related gene expression of Δ*vraR* treated with or without 10 μM PMAP-36 for 6 h. **F** SC19 and Δ*vraR* adhesion towards hCMEC/D3 cells after bacterial pre-incubation with or without 2.5 μM PMAP-36 for 6 h. **G** Representative images of Hoechst/PI double staining of RAW264.7 cells infected with SC19, Δ*vraS*, and Δ*vraR* where bacteria have been pretreated with or without 2.5 μM PMAP-36 for 6 h. Scale bar, 100 μm. **H** Quantification of PI-positive cells in (**G**) using ImageJ. All data are presented as mean ± SD and representative of three independent experiments with three biological replicates (*n* = 3). Statistical comparisons were performed using Student’s *t*-test for two groups and one-way ANOVA followed by Tukey’s post hoc test for three or more groups.

Given the critical role of the VraSR TCS in regulating *S. suis* antimicrobial resistance, we further investigate the regulatory role of VraSR in PMAP‑36-mediated *S. suis* adhesion and cytotoxicity using the Δ*vraS* and Δ*vraR* mutants. The results showed that the deletion of *vraS* and *vraR* alone did not affect *S. suis* growth, but PMAP-36 treatment delayed the growth of Δ*vraS* and Δ*vraR*, particularly inhibited their growth during the early growth phase from 0 to 3 h (Figure 2A, D)*.* Furthermore, PMAP-36 significantly downregulated the mRNA expression of CPS synthesis and adhesion-related genes in both Δ*vraS* and Δ*vraR* mutants (Figure 2B, E). In addition, the deletion of *vraS* and *vraR* tended to reduce the adhesive capacity of *S. suis*, although the decrease in the Δ*vraS* was not statistically significant (Figure 2C, F). Moreover, PMAP-36 treatment did not further alter the adhesion capacity of both the Δ*vraS* and Δ*vraR* mutants (Figure 2C, F)*.* Consistently, knockout of *vraS* and *vraR* significantly attenuated cytotoxicity towards murine macrophages, whereas PMAP-36 treatment exerted no additional effect on the cytotoxicity of the Δ*vraS* and Δ*vraR* mutants (Figure 2G, H)*.* These results demonstrate that the VraSR TCS is required for PMAP‑36-mediated enhancement of CPS/adhesion genes expression, bacterial adhesion, and cytotoxicity in *S. suis*.

### The VraS directly binds to PMAP-36

**Figure 3:**
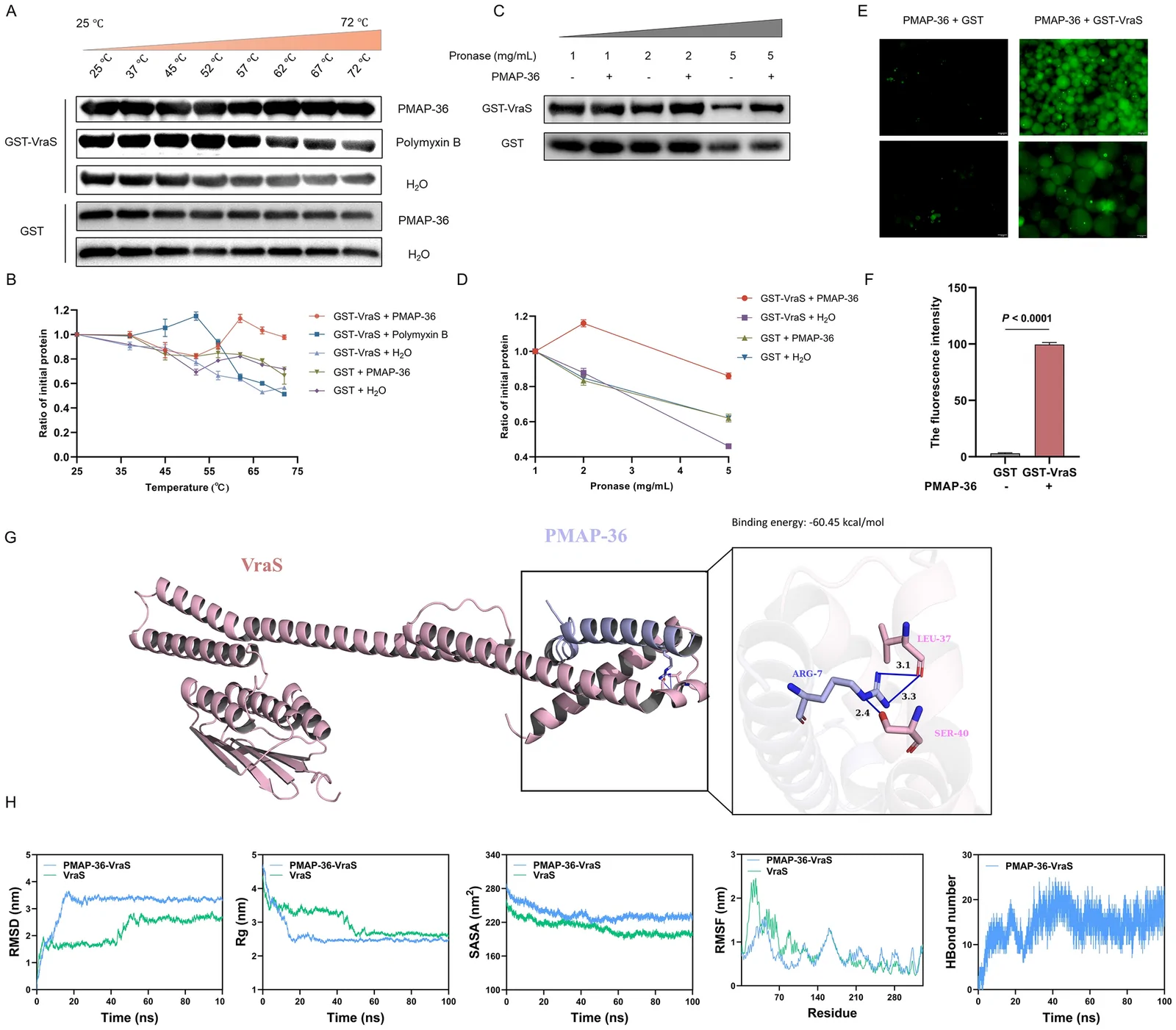
**The VraS directly binds to PMAP-36**. **A** Cellular thermal shift assay (CETSA) detecting the in vitro interaction between PMAP-36 and VraS. **B** Quantitative analysis of the CETSA result shown in (**A**). **C** Drug affinity responsive target stability (DARTS) assay showing the binding of PMAP-36 to VraS. **D** Quantitative analysis of the DARTS result shown in (**B**) using ImageJ. **E** GST affinity isolation assay detecting the in vitro interaction between PMAP-36 and VraS. Scale bar, 50 and 100 μm. **F** Quantitative analysis of the GST affinity isolation result shown in (**E**) using ImageJ. **G** Molecular docking between VraS and PMAP-36. **H** Molecular dynamics simulations. All data are presented as mean ± SD and representative of three independent experiments with three biological replicates (*n* = 3). Statistical analysis was performed using Student’s *t*-test.

To determine whether PMAP-36 directly interacts with VraS, we expressed and purified GST-tagged VraS protein to perform CETSA, DARTS, and GST affinity isolation assays. The results of CETSA showed that the thermal stability of VraS was not decreased in the presence of PMAP-36, as reflected by higher levels of nondegraded protein (Figure 3A, B). Notably, PMAP-36 and polymyxin B did not increase the thermal stability of only GST protein and VraS, respectively (Figure 3A, B), indicating the specific stability of PMAP-36 on VraS. In parallel, the DARTS assay showed that PMAP-36 protected VraS from proteolytic digestion, leading to significantly increased amounts of intact VraS protein, whereas GST alone still exhibited decreased trend in the presence of PMAP-36 (Figure 3C, D). Furthermore, a GST affinity isolation assay revealed that compared with the GST control group, the FITC-labeled PMAP-36 exhibited significantly stronger fluorescence intensity when incubated with GST-VraS fusion protein (Figure 3E, F), indicating direct binding between PMAP-36 and VraS. Structural modeling analysis further revealed the detailed interaction between VraS and PMAP-36 and predicted low binding energy (−60.45 kcal/mol) (Figure 3G). Molecular dynamics simulations analysis showed that the PMAP-36-VraS complex reached a stable conformational state, as indicated by the stable RMSD, Rg, SASA, and RMSF profiles, and sustained hydrogen-bond interactions (Figure 3H). These results demonstrate that VraS is a direct target of PMAP-36, providing a potential sensing basis for PMAP-36-mediated bacterial virulence.

### The VraSR TCS senses PMAP-36 to regulate fatty acid synthesis

**Figure 4:**
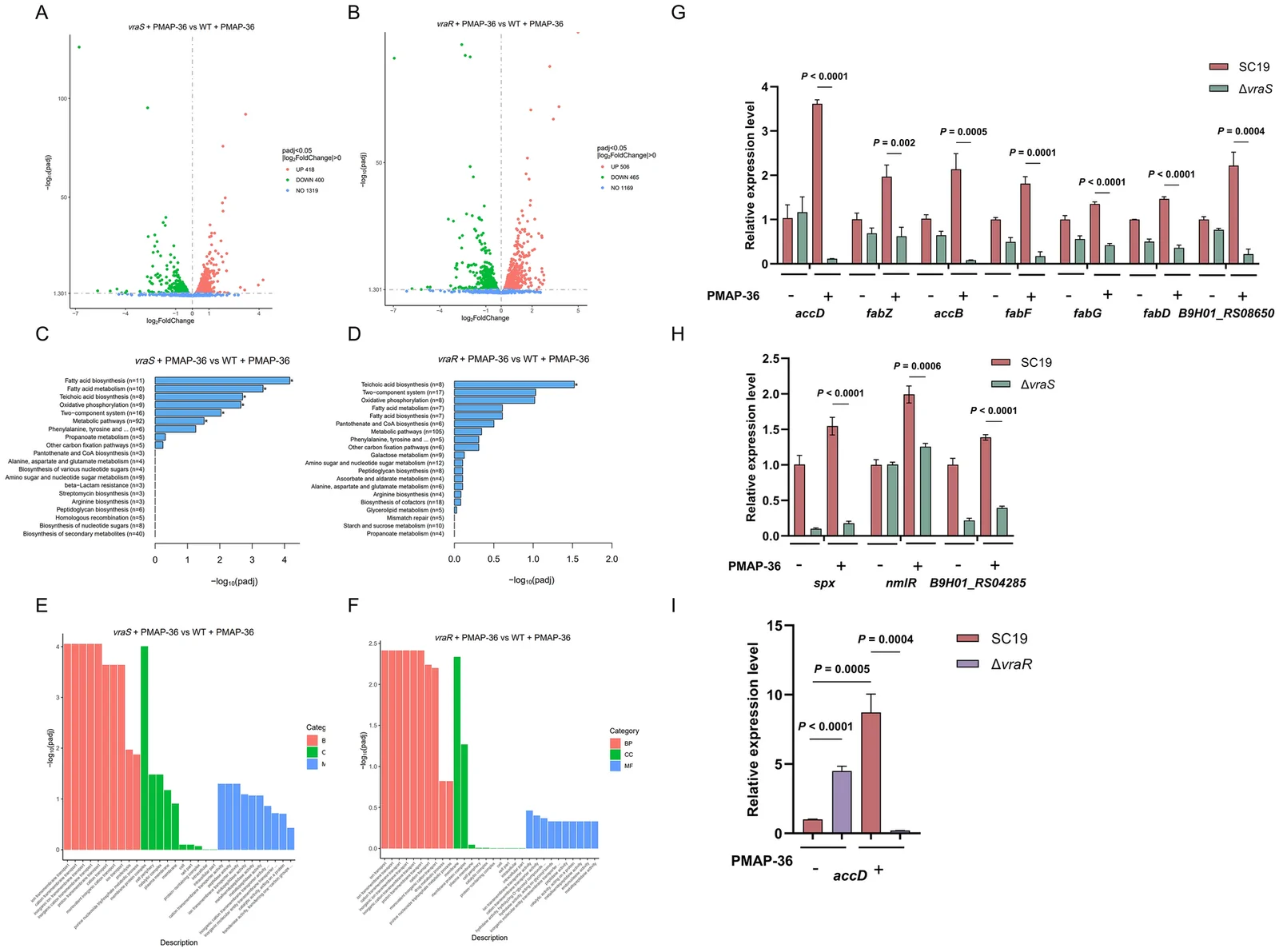
**Transcriptomic analysis of the VraSR TCS in sensing PMAP-36 to regulate fatty acid synthesis.** **A**, **B** Volcano plot of DEGs in WT SC19, Δ*vraS*, and Δ*vraR* strains treated with 10 μM PMAP-36 for 6 h. **C**, **D** Enriched KEGG pathway analysis of downregulated DEGs in Δ*vraS* and Δ*vraR* strains treated with 10 μM PMAP-36 for 6 h. **E**, **F** Enriched GO terms analysis of downregulated DEGs in Δ*vraS* and Δ*vraR* strains treated with 10 μM PMAP-36 for 6 h. **G** Fatty acid synthesis-related genes expression including *accD*, *fabZ*, *accB*, *fabF*, *fabG*, *fabD*, and *B9H01_RS08650* in SC19 and Δ*vraS* treated with or without 10 μM PMAP-36 for 6 h. **H** Oxidative stress-related genes expression of *spx*, *nmlR*, and *B9H01_RS04285* in SC19 and Δ*vraS* treated with or without 10 μM PMAP-36 for 6 h. **I** Gene *accD* expression in SC19 and Δ*vraR* treated with or without 10 μM PMAP-36 for 6 h. All data are presented as mean ± SD and representative of three independent experiments with three biological replicates (*n* = 3). Statistical analysis was performed using Student’s *t*-test.

To elucidate the molecular mechanism by which the VraSR TCS senses PMAP-36 to regulate *S. suis* virulence, transcriptomic analysis was performed to compare gene expression profiles among WT, Δ*vraS*, and Δ*vraR* strains under the treatment of PMAP-36. The results showed that compared with PMAP-36-treated WT strain, a total of 818 differentially expressed genes (DEGs) were identified in PMAP-36-treated Δ*vraS* mutant strain, including 418 upregulated and 400 downregulated DEGs (*P* < 0.05) (Figure 4A and Additional file 3). Similarly, PMAP-36-treated Δ*vraR* mutant strain exhibited 971 DEGs, including 506 upregulated and 465 downregulated DEGs (*P* < 0.05) (Figure 4B and Additional file 4). Kyoto Encyclopedia of Genes and Genomes (KEGG) pathway enrichment analysis revealed that these downregulated DEGs in both PMAP-36-treated Δ*vraS* and Δ*vraR* mutant strains were mainly enriched in fatty acid biosynthesis, fatty acid metabolism, teichoic acid biosynthesis, two-component system, and oxidative phosphorylation (Figure 4C, D), indicating that the VraSR TCS regulates cell membrane homeostasis, cell wall synthesis and stress adaptation in response to PMAP-36. Gene Ontology (GO) annotation analysis revealed that these downregulated DEGs in both PMAP-36-treated Δ*vraS* and Δ*vraR* mutant strains involved in biological process (BP), cellular component (CC), and molecular function (MF) were primarily enriched in transport, transmembrane transport, transporter activity, and amino acid biosynthesis (Figure 4E, F), indicating that VraSR mediates material transport and metabolic homeostasis in response to PMAP-36.

Next, we further verified the expression of downregulated DEGs in PMAP-36-treated Δ*vraS* mutant, which are in involved in significantly enriched in pathways including fatty acid biosynthesis and oxidative stress response. The results showed that PMAP-36 upregulated the expression of genes in fatty acid synthesis, including *accD*, *fabZ*, *accB*, *fabF*, *fabG*, *fabD*, and *B9H01_RS08650* as well as in oxidative stress response, including *spx*, *nmRl*, and *B9H01_RS04285* in WT strain (Figure 4G, H). However, PMAP-36 significantly downregulated these gene expression in Δ*vraS* mutant strain (Figure 4G, H). Among these DEGs in PMAP-36-treated Δ*vraS*, the gene *accD* was the most significantly downregulated, so we further verified its expression in Δ*vraR.* Interestingly, *vraR* deletion significantly upregulated *accD* expression but PMAP-36 remarkably downregulated its expression in Δ*vraR* (Figure 4I), indicating that the transcriptional regulation of *accD* is dependent on the VraR-mediated signaling pathway.

### Deletion of *accD* attenuates *S. suis* pathogenicity in mice infection model

**Figure 5:**
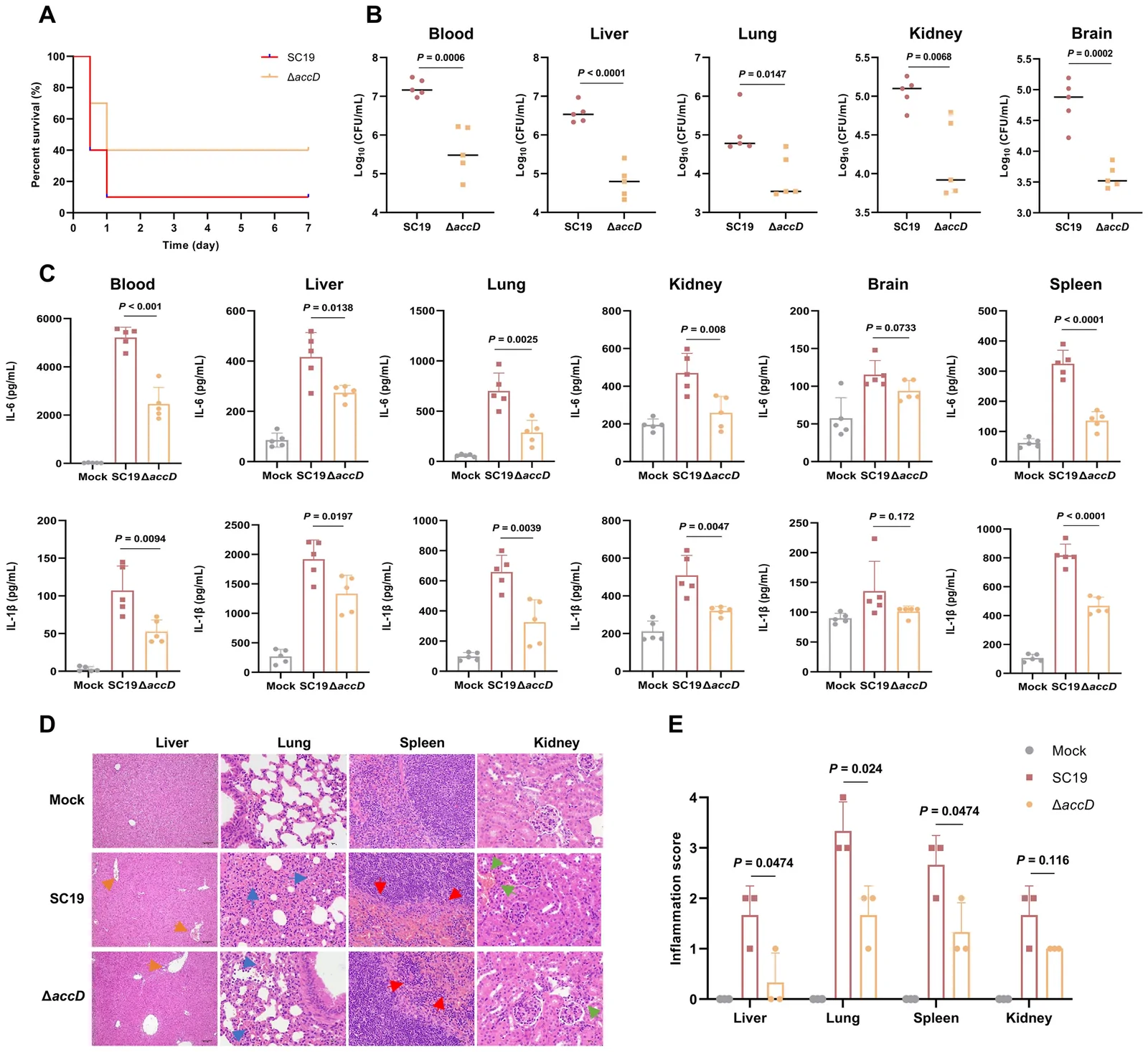
**Deletion of** ***accD*** **attenuates** ***S. suis*** **pathogenicity in mice infection model**. **A** Survival curves of mice (total *N =* 20, *n* = 10 per group) intraperitoneally challenged with SC19 or Δ*accD* (1.5 × 10^8^ CFU) and monitored for 7 d post-infection. **B** Bacterial burdens in blood and various organs of mice (total *N* = 10, *n* = 5 per group) at 10 h post infection. **C** IL-6 and IL-1β production in blood and different tissues (total *N =* 15, *n* = 5 per group). **D** Representative H&E-stained sections of liver, lung, spleen, and kidney (total *N =* 9, *n* = 3 per group). Scale bar, 10 μm. **E** Semi-quantitative inflammatory scores of lung, liver, spleen, and kidney. All data are presented as mean ± SD from 10, 5, or 3 biological replicates (*n* = 10, 5, or 3). Statistical analysis was performed using Student’s *t*-test.

Given that *accD* showed the most significant transcriptional alteration upon PMAP-36 exposure in VraSR-deficient strains and serves as a key functional gene in fatty acid biosynthesis, which is associated with bacterial membrane integrity, stress tolerance and pathogenicity, we generated Δ*accD* mutant strain to further investigate its effect on bacterial pathogenicity in mice infection model. The results showed that *accD* deficiency improved mice survival rate compared with WT strain (Figure 5A). In addition, bacterial loads in the blood, liver, lung, kidney, and brain were markedly reduced in Δ*accD*-infected mice compared with WT-infected mice (Figure 5B), indicating that *accD* deletion impairs in vivo colonization and attenuates virulence. Furthermore, WT SC19 infection induced high level production of IL-6 and IL-1β in the blood, liver, lung, kidney, brain, and spleen, whereas *accD* deletion significantly reduced cytokines production (Figure 5C). Histopathological examination revealed that WT SC19 induced severe organs damage, including interstitial pneumonia with alveolar capillary congestion and disruption of the pulmonary parenchyma in lung (Figure 5D, blue arrows), hepatic cord dissociation, sinusoidal dilatation and congestion accompanied by inflammatory cell infiltration in liver (Figure 5D, yellow arrows), septic splenitis in spleen (Figure 5D, red arrows), glomerular congestion, tubular injury, and interstitial nephritis in kidney (Figure 5D, green arrows). In contrast, Δ*accD*-infected mice exhibited alleviated pathological changes, including relatively intact alveolar structures with mild congestion in lung, normal architecture without obvious hepatic cord dissociation or edema in liver, reduced congestion, and intact white pulp lymphoid structures in spleen, and no significant glomerular or tubular degeneration with mild vascular congestion and negligible inflammatory infiltration in kidney (Figure 5D). Overall inflammation score analysis in these organs showed that *accD* deletion attenuated inflammation (Figure 5E). Collectively, these results demonstrate that *accD* deletion reduces SC19 pathogenicity in vivo.

### The VraSR-mediated *accD *expression regulates *S. suis* cytotoxicity and inflammatory response

**Figure 6:**
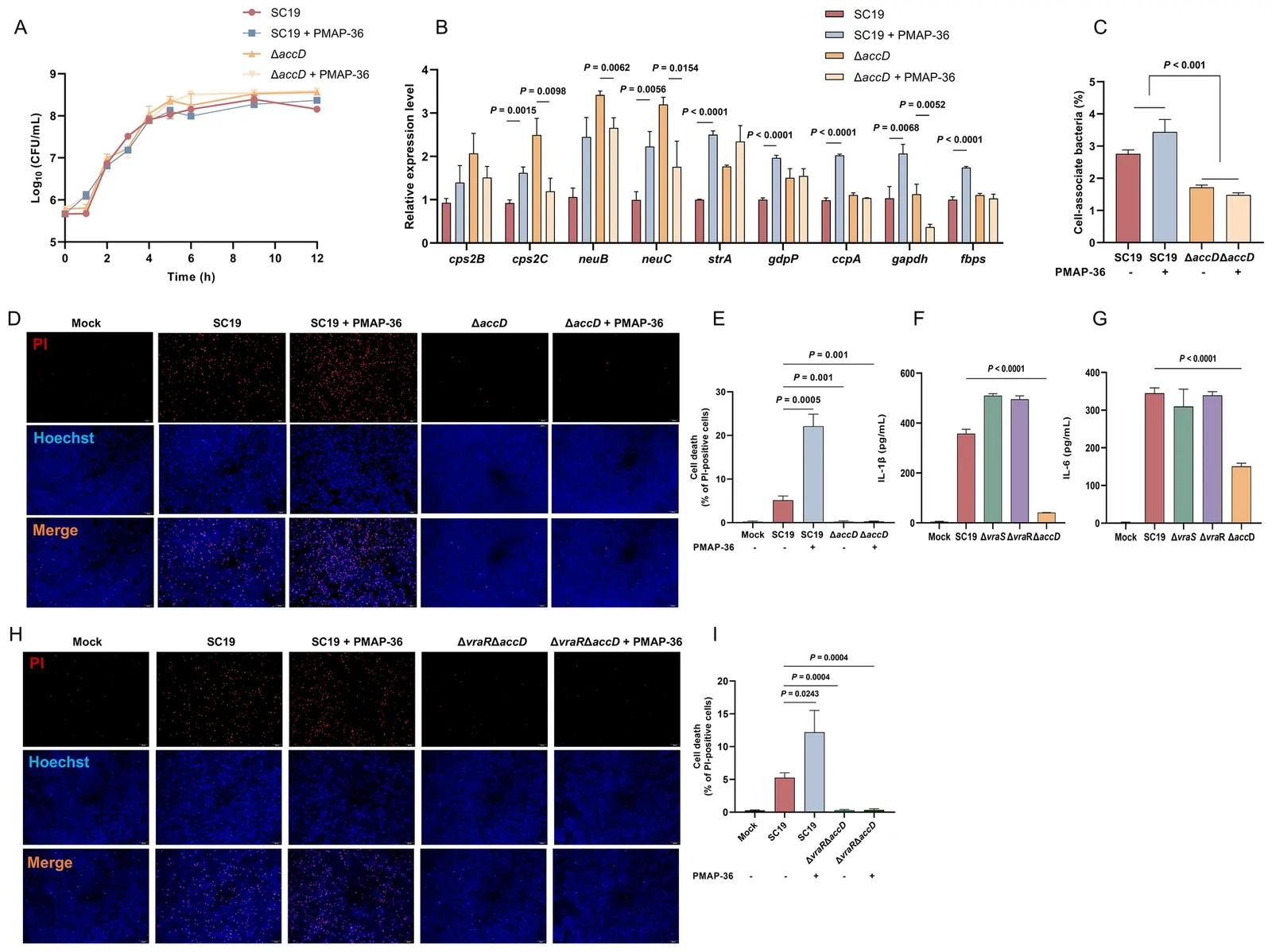
**The VraSR-mediated** ***accD*** **expression regulates** ***S. suis*** **cytotoxicity and inflammatory response**. **A** Growth curves of SC19 and Δ*accD* strains with or without 5 μM PMAP-36 treatment within 12 h. **B** CPS synthesis and adhesion-related gene expression of Δ*accD* treated with or without 10 μM PMAP-36 for 6 h. **C** SC19 and Δ*accD* adhesion towards hCMEC/D3 cells after bacterial pre-incubation with or without 2.5 μM PMAP-36 for 6 h. **D** Representative images of Hoechst/PI double staining of RAW264.7 cells infected with SC19 and Δ*accD* where bacteria have been pretreated with or without 2.5 μM PMAP-36 for 6 h. Scale bar, 100 μm. **E** Quantification of PI-positive cells in (**D**) using ImageJ. **F** Representative images of Hoechst/PI double staining of RAW264.7 cells infected with SC19 and Δ*vraR*Δ*accD* where bacteria have been pretreated with or without 2.5 μM PMAP-36 for 6 h. Scale bar, 100 μm. **G** Quantification of PI-positive cells in (**F**) using ImageJ. **H**, **I** Secretion levels of IL-6 (**H**) and IL-1β (**I**) in peritoneal macrophages infected with SC19, Δ*vraS*, Δ*vraR*, or Δ*accD*. All data are presented as mean ± SD and representative of three independent experiments with three biological replicates (*n* = 3). Statistical comparisons were performed using Student’s *t*-test for two groups and one-way ANOVA followed by Tukey’s post hoc test for three or more groups.

We found that *accD* deletion did not affect bacterial growth (Figure 6A). Knockout of *accD* upregulated expression of CPS synthesis genes including *cps2B*, *cps2C*, *neuB*, and *neuC*, as well as adhesion-related genes including *strA* and *gdpP*, but did not affect expression of adhesion-related genes including *ccpA*, *gapdh*, and *fbps* (Figure 6B). PMAP-36 downregulated expression of CPS synthesis genes, upregulated the expression of *strA*, downregulated the expression of *gapdh*, and did not affect the expression of *gdpP*, *ccpA* and *fbps* in Δ*accD* (Figure 6B), which reveals a critical role of *accD* on virulence genes expression in response to PMAP-36. In addition, *accD*-deficient strain exhibited decreased adhesion towards hCMEC/D3 cells, but PMAP-36 slightly reduced Δ*accD* adhesion (Figure 6C), indicating that PMAP-36 facilitates bacterial adhesion in an *accD*-dependent manner. Consistently, *accD* deficiency significantly decreased SC19-induced macrophage cell death (Figure 6D, E). In addition, *accD* deletion markedly decreased the secretion of pro-inflammatory cytokines IL-1β and IL-6 (Figure 6F, G). However, deletion of *vraS* or *vraR* failed to reduce cytokine production, which remained at high levels similar with those of the WT strain (Figure 6F, G). Notably, Δ*vraS* exhibited similar *accD* expression level with the WT strain, while Δ*vraR* displayed higher *accD* expression level compared with the WT strain (Figure 3G, I). Unsurprisingly, Δ*vraR*Δ*accD* displayed significantly reduced macrophage cell death (Figure 6H, I). These results demonstrate that VraSR-mediated *accD* expression contributes to *S. suis*-induced cytotoxicity and inflammatory response.

## Discussion

Bacterial TCSs are composed of a transmembrane sensor histidine kinase (HK) and an intracellular response regulator (RR), which are responsible for signaling transduction to regulate bacterial adaptability in response to extracellular stimuli, including AMPs, ions, temperature, pH, and oxygen [15]. Each TCS responds to a specific environmental signal, thus regulates virous biological process such as drug resistance, cellular homeostasis, and pathogenesis. To date, about ten groups of TCSs have been identified in *S. suis*, which are involved in virulence in mice or piglets through regulating virulence-associated genes, hemolytic activity, host cell adherence, capsule synthesis, survival in the phagocytic cells, and resistance to acidic and oxidative stress as well as drug resistance [15]. In particular, the VraSR and CiaRH have been identified in *S. suis* to regulate susceptibility to antibiotics or peptides including colistin, Polymyxin B and nisin [16, 17, 27]. This present study has shown that AMP PMAP-36 activated VraSR and subsequently upregulated virulence-associated genes expression as well as enhanced bacterial cytotoxicity to host cells. We identified the direct interaction between PMAP-36 and the TCS sensor full‑length VraS. This binding pattern also occurs in other pathogens, such as *Pseudomonas aeruginosa* (*P. aeruginosa*), *Vibrio cholerae* (*V. cholerae*) and *Staphylococcus aureus* (*S. aureus*) [13, 14, 28, 29], which full‑length membrane‑associated HK exhibits the highest binding affinity toward AMP. However, our study lacks the domain‑truncation mutagenesis to pinpoint the exact binding fragment. Therefore, further domain‑specific mutational work will help resolve this molecular detail in future investigations.

TCS HK sensing HDPs initiates the activation of TCS for signal transduction through autophosphorylation and inducing phosphorylation of RR, thereby regulating diverse genes expression to affect multiple bacterial virulent phenotypes or behaviors. Our study lacks the direct evidence of PMAP-36-mediated VraR phosphorylation, but the deletion of either *vraS* or *vraR* deletion significantly reduced PMAP-36-induced virulence/capsule synthesis/adhesion-associated genes expression, cell adherence and cell cytotoxicity, indicating that VraS coordinating with VraR regulates *S. suis* response to PMAP-36 and modulates its virulence phenotypes.

The interaction between HDPs and pathogenic bacteria has been extensively studied in the host immune system. In particular, the critical role of human cathelicidin LL-37 against bacterial infection has been well characterized. The loss of LL-37 aggravates bacterial infection in animal models. Conversely, numerous pathogens have evolved adaptive mechanisms to counteract HDP-mediated killing and numerous studies have reported that several TCSs are activated by LL-37 to promote bacterial pathogenesis. For instance, in *P. aeruginosa*, LL‑37 is sensed by the CprS sensor kinase, which triggers CprR‑dependent regulation of the type III secretion system via *higB* expression, ultimately enhancing macrophage cytotoxicity as indicated by increased LDH release [13]. In *V. cholerae*, LL-37 and human enteric α‑defensin 5 are sensed by CarS, leading to CarR phosphorylation and subsequent activation of *tcpP* transcription, which enhances bacterial adhesion and virulence gene expression [14]. Similarly, in *S. aureus*, LL‑37 activates the GraRS TCS, inducing bacterial tolerance to antibiotics and promoting peptidoglycan accumulation [29]. Although several factors from *S. suis*, including cysteine protease ApdS, endopeptidase O (PepO) and dipeptidylpeptidase IV, have been identify to cleave LL-37 and porcine AMP PR-39 to facilitate immune evasion [4, 5, 30], little is known about the interaction between porcine AMPs and *S. suis* as well as how *S. suis* TCS responds to porcine AMPs and facilitates its virulence. In our study, transcriptome profiling was performed in Δ*vraS* and Δ*vraR* mutants in response to PMAP-36 to identify VraSR-mediated virulence phenotypes. Our study revealed that the VraSR system of *S. suis* sensed porcine‑derived PMAP‑36 and regulated the expression of *accD*, a key gene involved in the initiation of fatty acid synthesis, thereby affecting bacterial adhesion and virulence genes expression. While the regulatory roles of VraSR in adhesion, capsule biosynthesis, and virulence have been extensively documented, this study for the first time identifies the porcine host-derived molecule PMAP-36 as an upstream activating signal of the VraSR system, and characterizes the *accD*-mediated fatty acid metabolic pathway as a novel downstream regulatory pathway of VraSR.

Fatty acid synthesis is essential for membrane formation and makes it an attractive antibacterial target for drug discovery [31]. A recent study has shown that the natural product emodin targets FabF involved in the elongation and synthesis of fatty acid to inhibit *S. suis* growth [32]. Fatty acid synthesis defect impairs biofilm stability, membrane integrity, bacterial adhesion, and virulence [33–35]. Based on the finding that PMAP-36 upregulated fatty acid synthesis-associated genes including *accD*, *accB*, *fabZ*, *fabF*, *fabG*, *fabD*, and *B9H01_RS08650*, our further study has shown that *accD* deletion significantly reduced *S. suis* pathogenicity in the mice infection model, as evidenced by decreased mortality, bacterial burden, and inflammatory response. Similar with FabG serving as an adhesin, *accD* has been identified to contribute to *S. suis* adhesion and the disruption of host cell membrane integrity. However, *accD* deletion significantly upregulated CPS synthesis-related genes, indicating a compensatory response to impaired fatty acid biosynthesis and disrupted cell membrane homeostasis, which is consistent with previous study that CPS deletion enhances *S. suis* adhesion [35, 36]. This represents a well‑documented phenotypic trade‑off in *streptococci* between capsule expression and bacterial adhesion. Elevated capsule production protects bacteria from host innate immune killing but typically masks surface adhesins and reduces adhesion and invasion. Interestingly, PMAP-36 treatment increased bacterial adhesion and upregulated *accD* expression, whereas deletion of *accD* resulted in increased expression of CPS-associated genes together with reduced adhesion, demonstrating that PMAP-36-mediated enhancement of bacterial adhesion is not only dependent on relieving capsule‑mediated masking of surface adhesins. VraSR probably coordinates additional downstream regulatory cascades that positively modulate bacterial adhesion, independent of capsule status. However, future work is required to separate direct VraSR‑mediated signaling events from indirect compensatory effects arising from disrupted fatty‑acid synthesis.

Notably, the transcription of adhesion-associated genes expression was not altered in the Δ*accD* mutant, demonstrating that the regulatory role of *accD* in bacterial adhesion and host membrane damage are independent of the transcriptional regulation of classical adhesion-related genes, and may rely on its essential function in maintaining bacterial membrane structure and fatty acid metabolism homeostasis. However, the exact mechanism by which *accD* regulates bacterial adhesion and CPS synthesis needs to be further studied. Our study lacks the direct evidence about VraSR-mediated the transcription of *accD*, but our data has shown that PMAP-36-upregulated *accD* was dependent on VraSR, indicating a central role of VraSR in regulating *accD* in response to PMAP-36. Notably, the residual regulatory effect of PMAP-36 on *accD* in the Δ*vraR* mutant suggests the existence of additional parallel regulatory mechanisms, which warrants further investigation in future work. On the other hand, a recent study in *S. aureus* has shown that the antimicrobial agent triclosan targeting FabI triggers bacterial membrane stress [37]. This stress is subsequently sensed by the SaeRS TCS, which in turn promotes bacterial virulence factors. Similarly, fatty acid metabolism has been also reported to regulate SaeRS activity to promote *S. aureus* infection [38]. In addition, the MtrAB TCS in *Mycobacterium abscessus* has been implicated to regulate fatty acid metabolism and biosynthesis [39]. These findings demonstrate that the interplay between TCSs and fatty acid metabolism represents a conserved and critical adaptive strategy exploited by diverse bacterial pathogens to sense environmental or host-derived stresses and coordinate pathogenicity.

In conclusion, our study demonstrates that the porcine HDP PMAP-36 is sensed by the VraSR TCS of *S. suis*, which in turn regulates the expression of the fatty acid synthesis key gene *accD* to promote bacterial pathogenicity. Specifically, *accD* knockout attenuated *S. suis* virulence in vivo, bacterial adhesion and host cell membrane damage. Our study provides new insights into targeting the VraSR-*accD* pathway as a novel strategy to prevent or treat *S. suis* infection.

## Supplementary Information

**Additional file 1.** **Target gene primers for mutant strains construction.**

**Additional file 2.** **Primers sequence for the detection of gene expression.**

**Additional file 3.** **Downregulated DEGs in ΔvraS treated with PMAP-36 compared with WT SC19 treated with PMAP-36.**

**Additional file 4.** **Downregulated DEGs in ΔvraR treated with PMAP-36 compared with WT SC19 treated with PMAP-36.**

## Data Availability

All data is available within the article and from the corresponding author on reasonable request. The raw data from RNA-Seq have been deposited in the NCBI Sequence Read Archive (SRA) database https://www.ncbi.nlm.nih.gov/bioproject/PRJNA1463217., the BioProject number is PRJNA1463217, and the sequence accession number is SRR38428966, SRR38428967, SRR38428968, SRR38428969, SRR38428970, SRR38428971, SRR38428972, SRR38428973, SRR38428974.

## References

[1] Mookherjee N, Anderson MA, Haagsman HP, Davidson DJ (2020) Antimicrobial host defence peptides: functions and clinical potential. Nat Rev Drug Discov 19:311–332 10.1038/s41573-019-0058-8 32107480

[2] Peng L, Tian H, Lu Y, Jia K, Ran J, Tao Q, Li G, Wan C, Ye C, Veldhuizen EJA, Chen H, Fang R (2022) Chicken cathelicidin-2 promotes NLRP3 inflammasome activation in macrophages. Vet Res 53:69 10.1186/s13567-022-01083-4 PMC9446576 36064470

[3] Xu L, Lu Y, Xu S, Liu Y, Liu H, Zhang T, Pan Y, Lu Y, Wang Z, Cao X, Li Z, Fang R, Peng L (2026) Cathelicidin CATH-2 suppresses the NF-κB/ROS/NLRP3 signaling pathway via regulating mTOR-dependent autophagy during *Streptococcus suis* infection. Vet Res 57:32 10.1186/s13567-025-01694-7 PMC12911090 41578407

[4] Xie F, Zan Y, Zhang Y, Zheng N, Yan Q, Zhang W, Zhang H, Jin M, Chen F, Zhang X, Liu S (2019) The cysteine protease ApdS from *Streptococcus suis* promotes evasion of innate immune defenses by cleaving the antimicrobial peptide cathelicidin LL-37. J Biol Chem 294:17962–17977 10.1074/jbc.RA119.009441 PMC6879338 31619521

[5] Jin M, Liang S, Wang J, Zhang H, Zhang Y, Zhang W, Liu S, Xie F (2023) Endopeptidase O promotes *Streptococcus suis* immune evasion by cleaving the host-defence peptide cathelicidins. Virulence 14:2283896 10.1080/21505594.2023.2283896 PMC10732652 38010345

[6] Jangir PK, Ogunlana L, MacLean RC (2021) Evolutionary constraints on the acquisition of antimicrobial peptide resistance in bacterial pathogens. Trends Microbiol 29:1058–1061 10.1016/j.tim.2021.03.007 33836929

[7] Joo HS, Fu CI, Otto M (2016) Bacterial strategies of resistance to antimicrobial peptides. Philos Trans R Soc Lond B Biol Sci 371:20150292 10.1098/rstb.2015.0292 PMC4874390 27160595

[8] Liao C, Liu Q, Luo G, Luo Y, Yao D, Wang Q, Zhang J, Wu Y, Jin J, Xu D, Lu W (2025) Human neutrophil α-defensin HNP1 interacts with bacterial OmpA to promote *Acinetobacter baumannii* biofilm formation. Nat Commun 16:5629 10.1038/s41467-025-60935-7 PMC12216189 40595622

[9] Xia X, Qin W, Zhu H, Wang X, Jiang J, Hu J (2019) How *Streptococcus suis* serotype 2 attempts to avoid attack by host immune defenses. J Microbiol Immunol Infect 52:516–525 10.1016/j.jmii.2019.03.003 30954397

[10] Xu J, Chen L, Pang S, Zhang Q, Deng S, Zhu J, Chen X, Langford PR, Huang Q, Zhou R, Li L (2024) HylS’, a fragment of truncated hyaluronidase of *Streptococcus suis*, contributes to immune evasion by interaction with host complement factor C3b. Virulence 15:2306691 10.1080/21505594.2024.2306691 PMC10854370 38251716

[11] Deng S, Liao J, Li H, Xu J, Fan J, Xia J, Wang J, Lei L, Chen M, Han Y, Zhai R, Zhou C, Zhou R, Cheng C, Song H (2024) *Streptococcus suis* subtilisin-like serine proteases SspA-1 and SspA-2 interplay with complement C3a and C5a to facilitate bacterial immune evasion and infection. Virulence 15:2301246 10.1080/21505594.2023.2301246 PMC10795781 38170683

[12] Xie H, Li Y, Fang Q, Xie H, Huang J, Wu Z, Deng Z, Sun Q, Huang Y, Liao J, Li S, Li Y, Fu Q (2025) SodA promotes immune evasion of *Streptococcus suis* by suppressing ROS accumulation and GSDMD-mediated mitochondrial disruption in neutrophils. Microbiol Spectr 14:e0190125 10.1128/spectrum.01901-25 PMC12772235 41294351

[13] Song Y, Zhang S, Zhao N, Nong C, He Y, Bao R (2024) *Pseudomonas aeruginosa* two-component system CprRS regulates HigBA expression and bacterial cytotoxicity in response to LL-37 stress. PLoS Pathog 20:e1011946 10.1371/journal.ppat.1011946 PMC10805311 38198506

[14] Liu Y, Xu T, Wang Q, Huang J, Zhu Y, Liu X, Liu R, Yang B, Zhou K (2022) *Vibrio cholerae* senses human enteric α-defensin 5 through a CarSR two-component system to promote bacterial pathogenicity. Commun Biol 5:559 10.1038/s42003-022-03525-3 PMC9178039 35676416

[15] Zheng C, Li L, Ge H, Meng H, Li Y, Bei W, Zhou X (2018) Role of two-component regulatory systems in the virulence of *Streptococcus suis*. Microbiol Res 214:123–128 10.1016/j.micres.2018.07.002 30031474

[16] Chang P, Li W, Shi G, Li H, Yang X, Xia Z, Ren Y, Li Z, Chen H, Bei W (2018) The VraSR regulatory system contributes to virulence in *Streptococcus suis* via resistance to innate immune defenses. Virulence 9:771–782 10.1080/21505594.2018.1428519 PMC5955479 29471718

[17] Zhong X, Zhang Y, Zhu Y, Dong W, Ma J, Pan Z, Roy S, Lu C, Yao H (2018) The two-component signaling system VraSRss is critical for multidrug resistance and full virulence in *Streptococcus suis* serotype 2. Infect Immun 86:e00096-18 10.1128/IAI.00096-18 PMC6013655 29685990

[18] Xu J, Fu S, Liu M, Xu Q, Bei W, Chen H, Tan C (2014) The two-component system NisK/NisR contributes to the virulence of *Streptococcus suis* serotype 2. Microbiol Res 169:541–546 10.1016/j.micres.2013.11.002 24342108

[19] Yuan F, Tan C, Liu Z, Yang K, Zhou D, Liu W, Duan Z, Guo R, Chen H, Tian Y, Bei W (2017) The 1910HK/RR two-component system is essential for the virulence of *Streptococcus suis* serotype 2. Microb Pathog 104:137–145 10.1016/j.micpath.2016.12.026 28093234

[20] Han H, Liu C, Wang Q, Xuan C, Zheng B, Tang J, Yan J, Zhang J, Li M, Cheng H, Lu G, Gao GF (2012) The two-component system Ihk/Irr contributes to the virulence of *Streptococcus suis* serotype 2 strain 05ZYH33 through alteration of the bacterial cell metabolism. Microbiology 158:1852–1866 10.1099/mic.0.057448-0 22504441

[21] Li J, Tan C, Zhou Y, Fu S, Hu L, Hu J, Chen H, Bei W (2011) The two-component regulatory system CiaRH contributes to the virulence of *Streptococcus suis* 2. Vet Microbiol 148:99–104 10.1016/j.vetmic.2010.08.005 20832201

[22] Li M, Wang C, Feng Y, Pan X, Cheng G, Wang J, Ge J, Zheng F, Cao M, Dong Y, Liu D, Wang J, Lin Y, Du H, Gao GF, Wang X, Hu F, Tang J (2008) SalK/SalR, a two-component signal transduction system, is essential for full virulence of highly invasive *Streptococcus suis* serotype 2. PLoS One 3:e2080 10.1371/journal.pone.0002080 PMC2358977 18461172

[23] Wang H, Shen X, Zhao Y, Wang M, Zhong Q, Chen T, Hu F, Li M (2012) Identification and proteome analysis of the two-component VirR/VirS system in epidemic *Streptococcus suis* serotype 2. FEMS Microbiol Lett 333:160–168 10.1111/j.1574-6968.2012.02611.x 22670712

[24] Lu Y, Xiang F, Xu L, Tian H, Tao Q, Jia K, Yin H, Ye C, Fang R, Peng L (2023) The protective role of chicken cathelicidin-1 against *Streptococcus suis* serotype 2 in vitro and in vivo. Vet Res 54:65 10.1186/s13567-023-01199-1 PMC10463303 37605242

[25] Li YH, Zhou YH, Ren YZ, Xu CG, Liu X, Liu B, Chen JQ, Ding WY, Zhao YL, Yang YB, Wang S, Liu D (2018) Inhibition of *Streptococcus suis* adhesion and biofilm formation in vitro by water extracts of* Rhizoma Coptidis*. Front Pharmacol 9:371 10.3389/fphar.2018.00371 PMC5911698 29713285

[26] Hu X, Lu Y, Yu X, Jia K, Xiong Q, Fang R (2024) The suppressive role of NLRP6 in host defense against *Streptococcus suis* infection. Vet Microbiol 296:110166 10.1016/j.vetmic.2024.110166 38968694

[27] Yang X, Peng W, Wang N, Dou B, Yang F, Chen H, Yuan F, Bei W (2022) Role of the two-component system CiaRH in the regulation of efflux pump SatAB and its correlation with fluoroquinolone susceptibility. Microbiol Spectr 10:e0041722 10.1128/spectrum.00417-22 PMC9241815 35638854

[28] Zhao NL, Zhu ZQ, Feng HZ, Song YJ, Huang Q, Mou XY, Nong C, He YX, Bao R (2023) Host-derived peptide signals regulate *Pseudomonas aeruginosa* virulence stress via the ParRS and CprRS two-component systems. J Hazard Mater 460:132512 10.1016/j.jhazmat.2023.132512 37703740

[29] Ledger EVK, Mesnage S, Edwards AM (2022) Human serum triggers antibiotic tolerance in *Staphylococcus aureus*. Nat Commun 13:2041 10.1038/s41467-022-29717-3 PMC9018823 35440121

[30] LeBel G, Vaillancourt K, Yi L, Gottschalk M, Grenier D (2018) Dipeptidylpeptidase IV of *Streptococcus suis* degrades the porcine antimicrobial peptide PR-39 and neutralizes its biological properties. Microb Pathog 122:200–206 10.1016/j.micpath.2018.06.029 29913265

[31] Yao J, Rock CO (2015) How bacterial pathogens eat host lipids: implications for the development of fatty acid synthesis therapeutics. J Biol Chem 290:5940–5946 10.1074/jbc.R114.636241 PMC4358231 25648887

[32] Wang J, Liu Y, Sun C, Chen L, Chen S, Huang X, Lu Z, Dong C, Zheng Y, Zhang Z, Liu Y, Qu Q, Li Y (2025) Effect of emodin on *Streptococcus suis* by targeting β-ketoacyl-acyl carrier protein synthase II. Phytomedicine 143:156821 10.1016/j.phymed.2025.156821 40339548

[33] Arjes HA, Gui H, Porter R, Atolia E, Peters JM, Gross C, Kearns DB, Huang KC (2022) Fatty acid synthesis knockdown promotes biofilm wrinkling and inhibits sporulation in *Bacillus**subtilis*. mBio 13:e0138822 10.1128/mbio.01388-22 PMC9600695 36069446

[34] Roszkowski EK, Charap S, Montague CR, Sukheja P, McNamara CW, Soma PS, Islam MN, Graham B, Grzegorzewicz AE, Jackson M, Yang B, Hay AG, Russell DG, Belisle JT, VanderVen BC (2026) Inhibition of *de novo* fatty acid synthesis in *Mycobacterium* tuberculosis. J Biol Chem 302:111022 10.1016/j.jbc.2025.111022 PMC12803843 41371338

[35] Guo G, Zhou Y, Li P, Li Q, Yu Y, Zhang W (2025) FabG moonlights as an extracellular adhesin mediates cytoadhesion of *Streptococcus suis* via interaction with plasminogen. BMC Microbiol 25:412 10.1186/s12866-025-04129-7 PMC12232145 40615777

[36] Okura M, Auger JP, Shibahara T, Goyette-Desjardins G, Van Calsteren MR, Maruyama F, Kawai M, Osaki M, Segura M, Gottschalk M, Takamatsu D (2021) Capsular polysaccharide switching in *Streptococcus suis* modulates host cell interactions and virulence. Sci Rep 11:6513 10.1038/s41598-021-85882-3 PMC7985379 33753801

[37] Boyd JM, Price EE, Roman Rodriguez F, Burchat N, Norambuena J, DuMont AL, Torres VJ, Sampath H (2025) Treatment of *Staphylococcus aureus* with environmentally relevant concentrations of triclosan activates SaeRS-dependent virulence factor expression. Antimicrob Agents Chemother 69:e0172824 10.1128/aac.01728-24 PMC12327003 40531055

[38] Yu J, Li M, Wang C, Hamushan M, Hu Y, Yang Y, Jiang F, Yan J, Wang B, Chen X, Tang J, Guo G, Han P, Shen H (2025) *Staphylococcus aureus* fatty acid metabolism governs *saeRS-*mediated aggregation in joint infections. Nat Commun 17:1152 10.1038/s41467-025-67910-2 PMC12859096 41423459

[39] Zhang J, Ju Y, Li L, Hameed HMA, Yusuf B, Gao Y, Fang C, Tian X, Ding J, Ma W, Chen X, Wang S, Zhang T (2025) MtrAB two-component system is crucial for the intrinsic resistance and virulence of *Mycobacterium**abscessus*. Int J Antimicrob Agents 65:107442 10.1016/j.ijantimicag.2024.107442 39761758

