# Supplementary material for: Two-component system VraSR senses host defense peptide PMAP-36 to promote Streptococcus suis pathogenicity through regulating accD expression
Source: Vet Res. 2026 Oct 22;57:185. doi: 10.1186/s13567-026-01858-z (PMC13693140; doi:10.1186/s13567-026-01858-z)
Supplement: Supplementary file 1 [file 13567_2026_1858_MOESM1_ESM.docx]

| gene name | | Primers sequence (5’-3’) | fragment length | restriction enzyme cutting site |
| --- | --- | --- | --- | --- |
| *vraS-SF* | CGCCAAGCTTTGATGTGGTAGCCAATGAGG | | 662bp | *Hind*Ⅲ |
| *vraS-SR* | ctcgaatcgtgcgaggagtaggtaggtacgtt | |  |  |
| *vraS-XF* | tactcctcgcacgattcgagtgatgctggt | | 759bp |  |
| *vraS-XR* | CGCGGATCCACACGTAGCGTGTCGTCAAT | |  | *BamH*I |
| *vraS-N1* | GAGGGAGAACGGAAACGGA | |  |  |
| *vraS-N2* | CTGCACCTTGTTGGAAGCC | |  |  |
| *vraR-SF* | CGCCAAGCTTtctatccactcttccgcagg | | 540bp | *Hind*Ⅲ |
| *vraR-SR* | CTCTTGCGCCCCAGCATCACTCGAATCGTAT | |  |  |
| *vraR-XF* | GTGATGCTGGGGCGCAAGAGGACCAATAG | |  |  |
| *vraR-XR* | | CGCGGATCCcccaacgaacactacgcaat | 609 | *BamH*I |
| *vraR-N1* | TGGTTATGCCCAAGATGACTG | |  |  |
| *vraR-N2* | CATATCCCTTGGCTAACAGCGT | |  |  |

Target gene primers for mutant strains construction
