# Supplementary material for: Two-component system VraSR senses host defense peptide PMAP-36 to promote Streptococcus suis pathogenicity through regulating accD expression
Source: Vet Res. 2026 Oct 22;57:185. doi: 10.1186/s13567-026-01858-z (PMC13693140; doi:10.1186/s13567-026-01858-z)
Supplement: Supplementary file 2 [file 13567_2026_1858_MOESM2_ESM.docx]

Primers sequence for the detection of gene expression

| gene name | | | | Forward primers | Reverse primers |
| --- | --- | --- | --- | --- | --- |
| *vraS* | | | | GAGGGAGAACGGAAACGGATTG | CCTGCGGAAGAGTGATAGATTGG |
| *vraR* | | | | TTCGAGTGATGCTGGTTGAC | TCTTGGGCATAACCAAGTCC |
| *salK* | | | | GGGGACTATTACTTTGAGCAAGCGATTG | CCTTCTCTCCTTCCACCATC |
| *salR* | | | | TCGCGTTCTGTCAATTCATC | TGACGGGATTTGATTTACCTG |
| *virR* | | | | GGGATTGCAGTGGAAATCAG | CATGGGAATGATTGGTCACA |
| *virS* | | | | GCAGCGAGATGTGACGGTAGC | GCGTGCCTGAGAGGATGAAACC |
| *yesM* | | | | ATACCAATCGCATCCGCATTCAAC | CCCTGTCCGTAATGTAGTCTGCTAG |
| *yesN* | | | | TCTCTCTAGCGGCAAACCAT | CCCTAGGGGCAATGGATTAT |
| *nisK* | | | | TTCTCTTTAAGGTTTCAGCGATGGC | TGGTTGCTTGCTCTTTCAATGTCC |
| *nisR* | | | CCTCCCCACTGTGTCTTGAT | | CAAGCAACCCGAGGAGAGTA |
| *cps2B* | | | CAAGGTAAGCGATGTGACGA | | ACACGGTCATCCAAAACCTC |
| *cps2C* | | | CGTCTTGGTGGATGCAGATA | | TTGGGAGAAACCTTTCCTGA |
| *neuB* | | | TAGCACAGATATGCCCGTTTAT | | ACAGCCATACCAGTTGAAAGA |
| *neuC* | | | TTTTGTGACAGGCTCTCGTG | | CGTATCCGTCAAATGCAATG |
| *strA* | | | AGGCGAAACAATTTCCACAC | | GGAGCTGGTACCATGAAGGA |
| *gdpP* | | CTTCTGCGATTGTCTGGTCA | | | AATTGAGGCGGTATTCGTTG |
| *ccpA* | TCTACAAGCGGCCCTGATAC | | | | AGACAAGATTCGTGCGGAGT |
| *gapdh* | GCTCCTGGTGGTAACGATGT | | | | ACCTTTTTGAACGCCAAATG |
| *fbps* | CAGTCGAGTGTGCGGTTAGA | | | | GGTGTCAACGGACTCCAGAT |
| *16s* | CTAGGATTTTCGTCTAAGTT | | | | TTGGTGATCAATAAACATGA |
